# Supplementary material for: Uncertainty quantification in the radiogenomics modeling of EGFR amplification in glioblastoma
Source: Sci Rep. 2021 Feb 16;11:3932. doi: 10.1038/s41598-021-83141-z (PMC7886858; doi:10.1038/s41598-021-83141-z)
Supplement: Supplementary file 1 — Supplementary Information [file 41598_2021_83141_MOESM1_ESM.pdf]

Uncertainty Quantification in the Radiogenomics modeling of EGFR Amplification in Glioblastoma

Leland S. Hu*[#], MD[1,2,3], Lujia Wang[#], MS[1,2,3], Andrea Hawkins-Daarud, PhD[3], Jennifer M. Eschbacher, MD[4], Kyle W. Singleton, PhD[3], Pamela R. Jackson, PhD[3], Kamala Clark-Swanson BS[3], Christopher P. Sereduk[9,11], Sen Peng[12], Panwen Wang[13], Junwen Wang[13], Leslie C. Baxter, PhD[5], Kris A. Smith, MD[6], Gina L. Mazza, PhD[7], Ashley M. Stokes, PhD[8], Bernard R. Bendok, MD[9], Richard S. Zimmerman, MD[9], Chandan Krishna, MD[9], Alyx B. Porter, MD[10], Maciej M. Mrugala, MD[10], PhD, Joseph M. Hoxworth, MD[1], Teresa Wu, PhD[1,2], Nhan L. Tran, PhD[9,11], Kristin R. Swanson[#], PhD[3,9], Jing Li[#], PhD[1,2,3]

*Corresponding author
# contributed equally

Affiliations:
1. Department of Radiology, Mayo Clinic Arizona, 5777 E. Mayo Blvd, Phoenix AZ, 85054
2. School of Computing, Informatics, and Decision Systems Engineering, Arizona State University, 699 S Mill Ave, Tempe, AZ, 85281, USA.
3. Mathematical NeuroOncology Lab, Precision Neurotherapeutics Innovation Program, Mayo Clinic Arizona, 5777 East Mayo Blvd, Support Services Building Suite 2-700, Phoenix, AZ, 85054.
4. Department of Pathology, Barrow Neurological Institute - St. Joseph's Hospital and Medical Center, Phoenix, AZ, 85013
5. Department of Neuropsychology, Mayo Clinic Arizona, 5777 E. Mayo Blvd, Phoenix AZ, 85054
6. Department of Neurosurgery, Barrow Neurological Institute - St. Joseph's Hospital and Medical Center, Phoenix, AZ, 85013
7. Department of Biostatistics, Mayo Clinic Arizona, 5777 E. Mayo Blvd, Phoenix AZ, 85054
8. Department of Imaging Research, Barrow Neurological Institute - St. Joseph's Hospital and Medical Center, Phoenix, AZ, 85013
9. Department of Neurosurgery, Mayo Clinic Arizona, 5777 E. Mayo Blvd, Phoenix AZ, 85054
10. Department of Neuro-Oncology, Mayo Clinic Arizona, 5777 E. Mayo Blvd, Phoenix AZ, 85054
11. Department of Cancer Biology, Mayo Clinic Arizona, 5777 E. Mayo Blvd, Phoenix AZ, 85054
12. Cancer and Cell Biology Division, Translational Genomics Research Institute, Phoenix, AZ 85004
13. Department of Health Sciences Research, Center for Individualized Medicine, Mayo Clinic, Scottsdale, AZ 85259

Funding: NS082609, CA221938, CA220378, NSF-1149602, Mayo Clinic Foundation, James S. McDonnell Foundation, Ivy Foundation, Arizona Biomedical Research Commission

Correspondence:

Leland S. Hu, M.D.
Department of Radiology
Mayo Clinic, Arizona
Hu.Leland@Mayo.Edu
Phone: (480) 301-7470
Fax: (480) 301-4303

Key words: Uncertainty, Radiogenomics, Glioblastoma, Glioma, Intratumoral, Heterogeneity

<u>Author contributions</u>:

LSH contributed to conception, design of the work, acquisition, analysis, and interpretation of data
LW contributed to conception, design of the work, acquisition, analysis, and interpretation of data
AHD contributed to analysis and interpretation of data
JME contributed to acquisition and analysis of data
KS contributed to acquisition and analysis of data
PRJ contributed to acquisition and analysis of data
KCS contributed to acquisition and analysis of data
CPS contributed to acquisition and analysis of data
SP contributed to acquisition and analysis of data
PW contributed to acquisition and analysis of data
JW contributed to acquisition and analysis of data
LCB contributed to design of the work and acquisition of data
KAS contributed to design of the work and acquisition of data
GM contributed to acquisition and analysis of data
AS contributed to acquisition and analysis of data
BRB contributed to analysis and interpretation of data
RSZ contributed to analysis and interpretation of data
CK contributed to analysis and interpretation of data
ABP contributed to analysis and interpretation of data
MMM contributed to analysis and interpretation of data
JMH contributed to analysis and interpretation of data
TW contributed to analysis and interpretation of data
NLT contributed to design of the work, acquisition, analysis, and interpretation of data
KRS to contributed conception, design of the work, acquisition, analysis, and interpretation of data
JL to contributed conception, design of the work, acquisition, analysis, and interpretation of data

Supplemental Table S1: **Patient demographics for the 25 untreated high-grade glioma tumors in the training set**. Patients labeled A through Y. M=male, F=female. Patient L on histology was diagnosed as Grade III Anaplastic Astrocytoma, although molecular features were consistent with Glioblastoma (e.g., EGFR amplification). NA = not available. Wt = wild type. aCGH = array comparative genomic hybridization; WES = whole exome sequencing

Patient	Type	Pathology	IDH.status	Sequenced
A	Glioblastoma	primary	NA	aCGH
B	Glioblastoma	primary	NA	aCGH
C	Glioblastoma	primary	NA	aCGH
D	Glioblastoma	primary	NA	aCGH
E	Glioblastoma	primary	NA	aCGH
F	Glioblastoma	primary	NA	aCGH
G	Glioblastoma	primary	NA	aCGH
H	Glioblastoma	primary	NA	aCGH
I	Glioblastoma	primary	NA	aCGH
J	Glioblastoma	primary	NA	aCGH
K	Glioblastoma	primary	NA	aCGH
L	Anaplastic Astrocytoma	primary	NA	aCGH
M	Glioblastoma	primary	NA	aCGH
N	Glioblastoma	primary	wt	WES

O	Glioblastoma	primary	wt	WES
P	Glioblastoma	primary	wt	WES
Q	Glioblastoma	primary	V178I/G105G	WES
R	Glioblastoma	primary	wt	WES
S	Glioblastoma	primary	R132H	WES
T	Glioblastoma	primary	wt	WES
U	Glioblastoma	primary	NA	aCGH
V	Glioblastoma	primary	wt	WES
W	Glioblastoma	primary	wt	WES
X	Glioblastoma	primary	V178I	WES
Y	Glioblastoma	primary	wt	WES

Supplemental Table S2: **Patient demographics for the 7 untreated high-grade glioma tumors in the validation set**. Patients labeled AA through GG. M=male, F=female. NA = not available. Wt = wild type. aCGH = array comparative genomic hybridization; WES = whole exome sequencing

Patient	Type	Pathology	IDH.status	Sequenced
AA	Anaplastic Astrocytoma	primary	WT	WES
AB	Anaplastic Astrocytoma	primary	WT	WES
AC	Glioblastoma	primary	WT	WES
AD	Anaplastic Astrocytoma	primary	WT	WES
AE	Glioblastoma	primary	WT	WES
AF	Glioblastoma	primary	WT	WES
AG	Anaplastic Astrocytoma	primary	WT	WES
